# The impact of sexual harassment on job satisfaction, turnover intentions, and absenteeism: findings from Pakistan compared to the United States

**DOI:** 10.1186/2193-1801-3-215

**Published:** 2014-05-01

**Authors:** Rebecca S Merkin, Muhammad Kamal Shah

**Affiliations:** Baruch College – CUNY, 1 Bernard Baruch Way, New York, N.Y. 10010 USA; AIR University, Islamabad, Pakistan

**Keywords:** Sexual harassment, Pakistan, Absenteeism, Turnover, Job satisfaction, Collectivism, Power distance

## Abstract

The purpose of this study was to compare and contrast how differences in perceptions of sexual harassment impact productive work environments for employees in Pakistan as compared to the US; in particular, how it affects job satisfaction, turnover, and/or absenteeism. This study analyzed employee responses in Pakistan (*n* = 146) and the United States (*n* = 102, 76) using questionnaire data. Significant results indicated that employees who were sexually harassed reported (a) a decrease in job satisfaction (b) greater turnover intentions and (c) a higher rate of absenteeism. Cross-cultural comparisons indicated that (a) Pakistani employees who were sexually harassed had greater job dissatisfaction and higher overall absenteeism than did their US counterparts and (b) Pakistani women were more likely to use indirect strategies to manage sexual harassment than were US targets.

Over the past few decades, there has been a growing acknowledgment of the significance of sexual harassment in the workplace. Researchers have found that sexual harassment experiences are negatively associated with job-related outcomes, psychological health, and physical health conditions (Chan et al. 2008). For example, sexual harassment affects women by undermining their job satisfaction and affective commitment (Shaffer et al. 2000; Shupe et al. 2002) and by undermining their well-being, increasing their psychological distress, causing greater physical illness, and causing greater disordered eating (Cortina et al. 2001; Gutek 1985; Hashmi, et al. 2013; Huerta et al. 2006). Moreover, both male and female employees’ well-being are diminished when they are working in an organizational context perceived as hostile toward women, even in the absence of personal hostility experiences (Miner-Rubino and Cortina 2004). Findings also show that workplace sexual harassment is responsible for psychological conditions such as stress, depression, and anxiety resulting in declines in organizational performance and productivity (Adams 1988; Baba et al. 1998).

Job outcomes are also affected by sexual harassment. Quick et al. (1992) found that sexual harassment negatively affects job involvement, job satisfaction, and organizational commitment (Shupe et al. 2002; Willness et al. 2007). Other outcomes such as tardiness and absenteeism (Baba et al. 1998; Jacobsen et al. 1996), sick leave and health compensation claims (Cartwright and Cooper 1997), and turnover intentions (Baba et al. 1998; Shaffer et al. 2000) are also evidenced as resulting from sexual harassment. Similarly, stress-related health problems such as heart disease, migraines, and ulcers are associated with employee productivity decreases (Adams 1988), such as disability claims (Gebhardt and Crump 1990) and organizational withdrawal behavior, including absenteeism, turnover, and early retirement (Hanisch and Hulin 1990).

Furthermore, sexual harassment is statistically related to general incivility and it tends to co-occur in organizations, leading to greater degenerated employee well-being with the addition of each type of workplace mistreatment (Lim and Cortina 2005). However, compared to bullying, sexual harassment has more adverse effects on health outcomes -- especially prominent among girls and sexual minorities (Gruber and Fineran 2008). On the other hand, victims of sexual harassment experience weaker adverse outcomes than victims of workplace aggression (Hershcovis and Barling 2010). Overall, women endure greater frequencies of incivility than men; nevertheless, both genders experience equally negative effects from sexual harassment in terms of job satisfaction, job withdrawal, and career salience (Cortina et al. 2001). Given the large number of harmful effects, sexual harassment merits serious consideration and warrants more in-depth examination.

Recent studies show that there are substantial differences in perceptions of sexual harassment in line with country of residence (Fiedler and Blanco 2006; Luthar and Luthar 2007; Matsumoto and Juang 2013; Tudor 2010; Zippel 2006). For example, in some countries there are women who are likely to tolerate sexual harassment for various cultural reasons and men who are more likely to sexually harass others (Cortina and Wasti 2005; Luthar and Luthar 2007; Wasti et al. 2000). Although it is often assumed by US Americans that sexual harassment affects work outcomes similarly in all cultures, this assumption has not been empirically tested. Consequently, this study considers whether the experience of sexual harassment impacts productive work environments for employees in Pakistan as well as the US; in particular, how it affects job satisfaction, turnover, and/or absenteeism.

In addition, differing cultural perceptions may lead to different modes of communication as a reaction to sexual harassment attempts. Preferences for differing reaction strategies have implications for managing sexual harassment in the workplace. Therefore, this investigation will also examine differing communication styles used by targets to handle sexual harassment. To accomplish this, this study will begin with the literature pertaining to US, then Pakistani sexual harassment, and followed by quantitative tests to measure cultural similarities and differences between sexual harassment in the US and Pakistan. Finally, an examination of communicative reactions to sexual harassment will be carried out.

## US sexual harassment definitions, laws, and perceptions

US courts acknowledge two types of sexual harassment based on the experiences of targets in the US. *Quid pro quo harassment* is when a supervisor requires sexual consideration from an employee in exchange for the granting or denial of employment benefits while *hostile work environment harassment* is when a reasonable woman perceives an abusive working environment (Rotundo et al. 2001). Targets of hostile work environment sexual harassment are required to prove that the harassment is amply severe or pervasive to alter the conditions of their employment by creating an abusive working environment, according to the Equal Employment Opportunity Commission (EEOC). The U.S. Merit System Protection Board (U. S. Merit Systems Protection Board 1981, 1987), developed a simple enumeration of seven harassing behaviors that were classified into three levels of severity: less severe (unwelcome sexual remarks. suggestive looks and gestures, and deliberate touching), moderately severe (pressure for dates, pressure for sexual favors, and unwelcome letters and telephone calls), and most severe (actual or attempted rape or sexual assault). Although sexual harassment laws are enacted in the US, the burden of proof falls on the target to present in court (O’Connor 1999).

In order to understand sexual harassment in Muslim majority countries, Syed (2008) points out that it might not be appropriate to apply a conventional equal-opportunity Western approach. Instead, he argues that it is necessary for gender equality discourses to consider local socio-cultural and economic considerations. Syed reasons that, while the disadvantages faced by women are in many ways universal, attempts to improve gender equality in employment may take diverse paths depending upon various contextual factors.

## Pakistani sexual harassment definitions, laws, and perceptions

Given that the socio-cultural, religious, and economic contexts within Western and Islamic contexts are substantially different (Syed 2008), Pakistani laws and their cultural enactment correspond in kind. Clear provisions exist in both Islam and the 1973 Pakistani Constitution to provide respect, safety, and equal rights for women; however, Pakistan remains a male-dominated culture where women still struggle to attain their rights (Akhtar and Métraux 2013). Though sexual harassment is not clearly defined in Pakistan, it accompanies other violent acts against women such as honor killing, acid throwing, bride burning, domestic violence, denial of property, rape, human trafficking, trafficking for forced labour and sex, child marriages, obscene phone calls, torture, and the exchange of females to settle disputes (Nosheen 2011). At times, Pakistani women are even suppressed and victimized by their own family members (Akhtar and Métraux 2013; Nosheen 2011).

Several amendments have been added to the Pakistani constitution to enhance political and economic rights for women, but the State does not apply its own laws to defend them (Akhtar and Métraux 2013; Nosheen 2011; Qureshi 2013). In fact, Pakistani women are frequently suppressed and victimized by law enforcement agencies such as the police (Abbas 2011). Moreover, female politicians, most notably Benazir Bhutto, have been killed when they raised their voices against fundamentalists and anti-female forces (Hall 2011).

Pakistani sexual harassment has become an issue that has received researchers’ attention particularly because of the growing number of women in the workforce (Saeed 2012). Pakistan is a traditional Muslim country characterized by patriarchy (Awan 2012; Latif 2009) and gender segregation (Yasin et al. 2010). On the one hand, the government has made continuous efforts to provide basic education to its citizens (Latif 2009). On the other hand, in Pakistan’s traditional society, with few links to the outside world, many girls are prohibited from getting an education and most women are kept at home or in low-paying jobs (Desai 1994; Nosheen 2011). Pakistani policies and laws on marriage and women’s education are described as methods of control to protect power and property (Akhtar and Métraux 2013). However, better internet access, an increased presence of global corporations, and a proliferation of television and radio stations, has increased Pakistanis’ awareness of how other societies treat women.

Nevertheless, Muslim societies such as Pakistan tend to dichotomize what they do from how they speak about themselves (Mernissi 1987). The first has to do with the realm of reality while the second has to do with identity (Mernissi 1987). Given the strong tendency within Muslim cultures to “save face” (Merkin and Ramadan 2010), what people say and what people do in Muslim societies tend to differ (Mernissi 1987). Therefore, opening up Pakistani society to women’s education and work may receive lip service but lukewarm efforts.

Recently, the Taliban shot a fourteen-year-old Pakistani girl, Malala, for encouraging girls to go to school (Turning-point 2012). Islamic fundamentalists believe that women’s education has destroyed the traditional boundaries and definitions of space and sex roles (Mernissi 1987). While globalization has propelled women to become educated and working, the male-dominant values of dismayed fundamentalists (Mernissi 1987) in Pakistani society reflect social and cultural values not religion (Akhtar and Métraux 2013). In fact, Islam allows woman to work outside the home in a job which suits her nature, especially when she or her family needs the outside work (Hifazatullah et al. 2011). Rather many Pakistani policies and laws on marriage and women’s education are cultural methods of control to protect power and property (Akhtar and Métraux 2013; Lakhvi and Suhaib 2010).

Pakistan’s inflationary economy is another reason women are activated to seek out jobs (Mangi 2011) despite gender imbalances that are the norm (Noureen and Awan 2011). With the female literacy rate at 47 percent (versus males which is at 67 percent), women always receive lower pay than their male counterparts in Pakistan (Yasin et al. 2010). Moreover, female work is not recognized and to a large extent is disregarded and invisible (Rives and Yousefi 1997). In Pakistan, traditional gender roles predominate and interaction between mixed dyads is prohibited (Sigal et al. 2005). Thus, when women enter into the public workplace, Pakistani men often make false assumptions that lead to sexual harassment. For example, Umul Awan, 25, who is an equity dealer at United Bank in Karachi, advises women, “You have to maintain your distance and create a stern image” (Mangi 2011).

In another split between what is said and what is done, the spokesperson for the Pakistan Nurses’ Association, Hameeda Bano, revealed that most patients and their relatives in Pakistan treat nurses as sex symbols; even though the country’s health minister, announced that the government is taking measures to improve the salaries and education of nurses (Yusufzai 2006). Not surprisingly, Pakistan still has an astounding nursing shortage. Specifically, Pakistan has 28,000 nurses to care for its 150 million people (Yusufzai 2006). In contrast, the United Kingdom has around 20 times as many nurses for its 60 million people while the US has the lowest nurse to patients ratios of 12 European countries (including the United Kingdom) besides for Norway (Aiken et al. 2012). Bano also pointed out that sexual assault is a problem in Pakistani hospitals. For example, one nurse was stabbed in the back when she refused an invitation to spend the night with a patient (Yusufzai 2006).

Thus, although Pakistan has made some strides in women’s education, cultural paternalism and inadequate legal protection from sexual harassment threaten women’s participation in the workforce (Raza 2007). In order for the Pakistani economy to attain sustainable economic growth, more women are needed to participate in the country’s workforce (Qureshi et al. 2007). Consequently, this study contributes to the sexual harassment literature by examining whether the experience of sexual harassment impacts productive work environments for employees in Pakistan as well as the US; in particular, how it affects job satisfaction, turnover, and/or absenteeism.

## Sexual harassment

### Sexual harassment and job satisfaction

US findings show that sexual harassment decreases job satisfaction (Antecol et al. 2009). Moreover, even if they are not personally being harassed, employees working in an organizational context perceived as hostile toward women also experience diminished well-being (Miner-Rubino and Cortina 2004). In addition to job satisfaction, sexual harassment has been found to negatively impinge on job involvement and organizational commitment (Shupe et al. 2002).

The life circumstances of Pakistani women present them with a dilemma because while they need to work for economic reasons, it is also assumed that they are culturally mandated to stay at home and only men are culturally viewed as the family breadwinners (Noureen and Awan 2011). Consequently, it is not surprising that findings show women’s job satisfaction is higher when they have a more balanced work and family life (Malik et al. 2010a).

Additionally, if Pakistani women do have support from family for being employed outside the home, they still face challenges from dominant males in the workplace (Mangi 2011). Thus, Pakistani data show that working conditions are an important predictor of organizational commitment and job satisfaction (Ahmed and Islam 2011). What is more, given women’s difficulty in achieving their desired status in Pakistani society (Ud Din et al. 2011), it is understandable why studies indicate that job satisfaction is highest in Pakistan when employees experience open communication and supervisory support (Khan et al. 2011). Despite the abundant literature on US job satisfaction indicating that sexual harassment in the US reduces job satisfaction (e.g., Antecol et al. 2009), little is known about the nature of responses to sexual harassment in Pakistan. To test the overall relationship between sexual harassment and job satisfaction, the following hypothesis is posed:

H1: Employees experiencing sexual harassment will have lower job satisfaction than employees not experiencing sexual harassment.

### Sexual harassment and turnover intentions

Job satisfaction tends to have an inverse relationship with turnover intentions (e.g., Amah 2009). Thus, if employees are not satisfied with their job, they are likely to have higher turnover intentions. Sexual harassment reduces job satisfaction by infiltrating the work environment of targets and observers alike (O’Leary-Kelly et al. 2009). Findings in Pakistan show that there is a negative correlation between an organizational environment, job satisfaction of employees, and turnover intentions (Shahzad et al. 2011). Thus, sexual harassment is likely to increase turnover, the cost of which is tremendous -- so tremendous that US findings show turnover costs are the largest single component of the overall cost of sexual harassment (Faley et al. 1994).

Evidence from US findings indicates that sexual harassment increases turnover intentions at work (Rosen and Martin 1998). Moreover, studies in the military show that sexually harassing incidents lead to increased actual turnover, even after controlling for job satisfaction (Sims et al. 2005). In addition, when women perceive that they have managerial support for the distress related to experienced sexual harassment, they had fewer turnover intentions (Brough and Frame 2004). Unfortunately, however, sexual harassment negatively predicts managerial support (Brough and Frame 2004). The following hypothesis tests the US as a baseline and the previously unexplored Pakistani population:

H2: Employees experiencing sexual harassment will have greater turnover intentions than employees who are not experiencing sexual harassment.

### Sexual harassment and absenteeism

In the past, Waters and Roach (1979) found turnover to be correlated with absenteeism. Findings also show that lower job satisfaction is associated with higher employee turnover and absenteeism (Hackman and Oldham 1975). Because US findings show that sexual harassment results in lower job satisfaction (e.g., O’Leary-Kelly et al. 2009), it is likely that sexual harassment would result in greater absenteeism as well. Sexual harassment, often leads to greater absenteeism and a loss of productive work in US studies (Kokubun 2007).

Similarly, findings in Pakistan indicate that higher job stress and lower job satisfaction result in greater absenteeism (Shahzad et al. 2011). Thus, the impact of sexual harassment should result in greater absenteeism in Pakistan as well because when work expectations are not met, absenteeism goes up (Patton 2008). This is also likely to be the case in Pakistan where findings show, for example, that employees who felt their salaries were lower than other employees despite their higher qualifications were also discouraged and increased their absenteeism (Habib 2010). To test this relationship, the following hypothesis is posed:

H3: Employees experiencing sexual harassment will have greater overall absenteeism than employees who are not experiencing sexual harassment.

### Cross-cultural differences and sexual harassment outcomes

#### Sexual harassment and employment outcomes in Pakistan versus the US

Work conditions in developing nations are much worse than they are in developed countries (Kortum et al. 2010). In Pakistan there are fewer opportunities for employees with higher qualifications, less personal safety at work, and poorer overall working conditions than in the US (Malik et al. 2010b). Pakistani women employees also indicate that they are discontented because their work arrangements do not adequately accommodate their personal and family needs (Faisal 2010).

For many years sexual harassment was not taken into account in Pakistan because there were no laws against it. Recently, however, the Protection Against Harassment of Women at Workplace Bill (2010) was passed in Pakistan to make sexual harassment illegal. The intent of this bill is to create a better working environment for women (i.e., free from harassment, abuse, and intimidation). Yet, despite Pakistan’s anti-sexual harassment law, Pakistani working women still experience rampant sexual harassment (Mangi 2011; Noureen and Awan 2011; Savitha 2010). This is partly because many Pakistanis do not see sexual harassment as a serious social issue and to some extent deny its presence. Due to a lack of support mechanisms, targets of sexual harassment in Pakistan are, to a large extent, ignored (Savitha 2010).

Few studies have been conducted on sexual harassment in developing countries. However, one finding from Turkey indicates that sexual harassment of female nurses remains a disturbing problem there (Kisa and Dziegielewski 1996). Findings indicate that sexual harassment remains a problem in Pakistan as well (Mangi 2011). Given the disparity in working conditions between the Pakistan and the US, the following hypotheses are posed:

H4: Employees experiencing sexual harassment will have lower overall job satisfaction in Pakistan than in the US.

H5: Employees experiencing workplace sexual harassment will have higher turnover intentions in Pakistan than in the US.

H6: Employees experiencing sexual harassment will have higher overall absenteeism in Pakistan than in the US.

### Sexual harassment and indirect strategies

#### Individualism and collectivism

Luthar and Luthar (2007) posit that Hofstede’s (1980) theory of cultural dimensions is a good starting point for investigating and understanding sexual harassment in a cross-cultural context. Despite the various criticisms of Hofstede’s study (e.g., McSweeney 2002), his rankings have heuristic value (Sigal et al. 2005). Moreover, Hofstede’s model has been validated in replication studies (e.g., Vishwanath 2003). What’s more, Hofstede’s model continues to influence cross-cultural sexual harassment literature (Fiedler and Blanco 2006). Thus, Hofstede’s dimensions of power distance and individualism/collectivism, the most relevant cultural dimensions to the discussion of sexual harassment (Cox et al. 2005), will be considered in the further analysis of whether direct or indirect response strategies are likely to be preferred to manage sexual harassment. Power distance refers to the extent to which less powerful individuals from a society accept inequality in power and consider it normal (Stohl 1993). Hofstede (1980) elucidates that individualistic cultures stress individual goals, whereas collectivistic cultures stress group goals.

Pakistan is high in power distance and a collectivistic culture (Sigal et al. 2005) while the US is low in power distance and an individualistic culture (Hofstede 1980). Findings indicate that there is a greater tolerance of sexual harassment and a higher likelihood to sexually harass in high power-distant cultures which are also collectivistic than in low power-distant countries which are also individualistic (Luthar and Luthar 2007).

Characteristics of collectivistic and high power-distant cultures are also congruent with paternalism (Aycan 2006). In patriarchal paternalistic societies, gender roles, honor, and shame codes reflect asymmetrical standards for women’s and men’s sexual behavior such as rewarding men but reproving women for early initiation into sexual life, numerous sex partners, and extramarital relationships (Cortina and Wasti 2005). In fact, in paternalistic societies like Pakistan, decisions are made by the husband or father in most families (Kovarik 2005). Prevalent paternal attitudes in Pakistan, such as shame brought upon a family if the father or husband lives off the livelihood of a wife or daughter, are responsible for many qualified women staying away from working (Kazim 2007; Naqvi et al. 2007). Moreover, those women who do decide to work in Pakistan are likely to encounter rampant sexual harassment (Savitha 2010) because cultural factors such as paternalism underlie the perceptions many Pakistanis have that sexual harassment is not a serious social issue and deny its presence (Morley et al. 2005).

Collectivistic values in Pakistan militate against a civil human rights approach to handling sexual harassment, owing to the focus on what is perceived as community interests as opposed to individual autonomy (Critelli 2010). Due to a lack of support mechanisms, Pakistani targets of sexual harassment are ignored, keep silent, and feel guilty about their experiences with sexual harassment (Morley et al. 2005). What’s more, because traditional gender roles predominate in Pakistan, women are likely to be discouraged from directly confronting the harasser. For example, among Pakistani medical students 83% of harassed women did not report it to authorities (Hashmi et al. 2013). One study shows that in high power-distant and collectivistic Turkey, women are inclined to manage sexual harassment with indirect nonverbal forms of communication (Wasti and Cortina 2002). A study in high-power-distance and collective Hong Kong also showed that women managers who are sexually harassed first try to avoid the harasser, then apply for a transfer or quit rather than confront the harasser or report the case to superiors (Chan et al. 1999). This is because collectivists believe that direct confrontations could possibly lead to a loss of face (Merkin et al. 2013).

In collective high-power-distant cultures, people are much more concerned with saving face and would, therefore, avoid direct communication strategies in response to sexual harassment, if at all possible, to preserve social harmony (Ino and Glicken 2002). On the other hand, in low-power-distant and individualistic cultures, direct communication is preferred even if it threatens the relationship (Merkin and Ramadan 2010). Thus, the following hypothesis is posed:

H7: Employees from Pakistan will respond less directly to sexual harassment than employees from the US.

## Method

### Participants

The Pakistani sample (*n* = 146) were health-care workers who came from the cities of Islamabad, Rawalpindi, Lahore and Karachi. Participants were 75% women (n = 110) and 25% men (n = 36). Overall, the Pakistani sample was young, urban, and educated. US participants (*n* = 102) were students in an urban New York university. US participants were 63% female (n = 64) and 37% male (n = 38). A second study was carried out between the same Pakistani sample and US health care workers (*n* = 96) as a control for the different sample types. The health care US participants were 77% female (n = 74) and 19% male (n = 22). For more demographic details, see Tables 1 and 2.

**Table 1:** **Operationalization of variables**

Variable name	Variable description
Sexual harassment	Sexual Experiences Questionnaire (SEQ)
Turnover intentions	Have you been trying to change your main occupation for the past 12 months?
Overall absenteeism	Sum of Absenteeism Illness and Absenteeism Stress
Absenteeism_Illness	In the past two years, have you taken off from work for more than a week due to work-related illness?
Absenteeism_Stress	In the past two years, have you taken off from work for more than a week due to work-related stress?
Job satisfaction	In general, how would you classify your degree of satisfaction with your present job? (From 1 = Very Satisfied to 5 = Very Dissatisfied).
	Wage level________
	Non-wage Benefits________
	Nature of work________
	Opportunity for improving skills________
	Opportunity for promotion________
	Work environment________
Age	What is your age?______
Education	What is your highest degree of schooling? (1 = none, 2 = elementary incomplete, 3 = elementary completed, 4 = high school incomplete, 5 = high school completed, 6 = college incomplete, 7 = college completed, 8 = Master or Doctoral degree completed.
Marital status	What is your marital status? 1 = single, 2 = Married, 3 = separated, 4 = divorced, 5 = widowed

*Note.* The six job satisfaction items were pooled to measure job satisfaction.

**Table 2:** **Demographic variables**

	United States	United States	Pakistani			
	Employed	Healthcare	Sample			
	Student sample	Sample				
Demographic variables	M	SD	M	SD	M	SD
Age	21	2.98	33	13.88	31	5.15
Education	6	.17	6.3	.86	7	1.03
Marital status	1.07	.26	1.65	1.06	1.65	.48

My institutional review board at Baruch College - CUNY approved my US data collection and M. Kamal Shah's data collected was approved by his AIR University Master's Program.

### Design and statistical procedures

Respondents filled out questionnaire items in their respective home countries. Given that education can bring extraordinary transformations in women’s lives by enhancing their confidence and raising their status (Noureen and Awan 2011), education could be a competing predictor with sexual harassment outcomes analyzed in this study. Thus, education was inserted as a covariate in the analyses that follow.

Hypotheses 1 through 3 were tested by separate regression analyses with sexual harassment and educational level as the independent variables and job satisfaction, turnover intentions, and absenteeism as the dependent variables. Hypotheses 4 through 6 were tested by means of a MANCOVA design with sexual harassment and country as the independent variables, education as the covariate, and job satisfaction, turnover intentions and absenteeism as the dependent variables. To test H7 regarding indirect responses to sexual harassment between the US and Pakistan, participants were requested to read the following vignette representing a sexually harassing scenario: “Imagine that you are working at the office and a superior asks you to make him or her coffee. Then this acquaintance invites you out for dinner. You decide to go. While dining in a fancy restaurant this superior asks you to come home with him/her. You don’t want to. What would you do?” Then they rated the provided indirect versus direct strategies in terms of likelihood of use. Respondents were asked to respond to the questionnaire items on a 5-point Likert scale ranging from (1) *strongly disagree* to (5) *strongly agree*. Then a regression analysis was conducted with country and education as the independent variables and indirect strategies as the dependent variable.

### Consent

Informed consent from the respondents for the publication of this report.

### Instrumentation

#### Culture

As suggested by Hofstede (1994), cultural dimensions were operationalized by country.

#### Sexual harassment

*Sexual harassment* was measured using the Sexual Experiences Questionnaire (SEQ; Fitzgerald et al. 1988; as revised by Fitzgerald et al. (1995), which has been described as a behaviorally based scale with excellent psychometric properties (Arvey and Cavanaugh 1995). The SEQ assessed the frequency with which women had experienced *gender harassment* (behaviors that convey sexist, degrading, and misogynistic attitudes about women) and *unwanted sexual attention* (unwanted touching, stroking, or repeated unwanted requests for romantic or sexual relationship). Participants reported the frequency with which they had experienced these behaviors from male coworkers or supervisors in the previous 2 years, using a 5-point scale with *1 = Never, 2 = Once, 3 = Some, 4 = Often, 5 = Most of the time*).

#### Job satisfaction, turnover intentions, overall absenteeism, and education

The operationalization of the dependent variables included (a) job satisfaction, which included job satisfaction due to wages, benefits, nature of work, improving skills, opportunity for promotion, and work environment (b) turnover intentions (c) overall absenteeism, which consisted of combined absenteeism due to stress and absenteeism due to illness items. For specifics of the control variable, education, and of other demographic questions, see Table 1. Reliabilities for job-outcome variables can be found in Table 3.

**Table 3:** **Reliabilities for job-outcome dependent variables**

	United States	Pakistan	Combined
Dependent variable	Reliability	Reliability	Reliability
Study 1			
Sexual harassment	.89	.93	.91
Overall absenteeism	.79	.73	.85
Job satisfaction	.83	.83	.82
Study 2			
Sexual harassment	.82		
Overall absenteeism	.86		
Job satisfaction	.86		

#### Indirect communication

This study employed Cocroft’s (1992) construction of response items for (in)direct strategies (e.g., I would try to express my regrets indirectly) because Cocroft and Ting-Toomey (1994) previously found these response items to be reliable and valid. Indirect reliability coefficients ranged from .76 (US Study 2) to .77 (US Study 1) and .77 (Pakistan) and averaged .77 across both groups.

## Results

### Sexual harassment and job satisfaction

H1 positing that employees experiencing sexual harassment will have lower overall job satisfaction than employees who are not experiencing sexual harassment was supported (*R*^*2*^ = .06; *F*(2, 107) = 3.33, *p* = .04) in the overall model. While sexual harassment (*t*(210) = −2.58, *p* = .01) explained job satisfaction levels in the US and Pakistan, education (*t*(210) = −.97, *p* = .331) was an insignificant predictor. In study 2, sexual harassment (*t*(158) = −1.74, *p* = .08) and education (*t*(158) = −.67, *p* = .51) were both insignificant predictors of job satisfaction levels in the US and Pakistan.

### Sexual harassment and turnover intentions

Regression analysis results (*t*(210) = 2.05, *p* = .007) substantiated H2 that employees from both the US and Pakistan experiencing sexual harassment would have greater turnover intentions (*R*^2^ = .05, *F*(2, 210) = 5.03, *p* = .04). Interestingly, the education covariate was also significant (*t*(210) = 2.72, *p* = .007) showing that education level impacts turnover intentions. In study 2, regression analysis results (*t*(158) = 2.55, *p* = .01) substantiated H2 that employees from both the US and Pakistan experiencing sexual harassment would have greater turnover intentions (*R*^*2*^ = .04, *F*(2, 158) = 3.31, *p* = .04). The education covariate in Study 2 was insignificant (*t*(158) = .42, *p* = .67).

### Sexual harassment and overall absenteeism

H3 that employees experiencing sexual harassment will have greater overall absenteeism than employees who are not experiencing sexual harassment was partially supported (*R*^2^ = .08; *F*(2, 210) = 9.14, *p* = .0001) in that the overall model with education was significant. However, education (*t*(210) = 4.21, *p* = .0001) was a more important predictor of absenteeism than sexual harassment (*t*(210) = 1.43, *p* = .156) which lost its significance after adding education to the regression model in Study 1. Study 2 had insignificant overall results for both predictors (*R*^*2*^ = .005; *F*(2, 158) = .38, *p* = .69)

### Sexual harassment, country, education, and job satisfaction

Overall results showed that multivariate analysis was warranted because the multivariate main effect for culture was significant (Wilks’ λ = .94; *F*(3, 203) = 4.35, *p* < .005, *ή2* = .06). Overall results for Study 2 also showed that multivariate analysis was warranted because the multivariate main effect for culture was significant (Wilks’ λ = .77; *F*(3, 140) = 13.59, *p* < .0001*, ή2* = .23). In the MANCOVA test, education had insignificant effects and was therefore, dropped from further analysis. Univariate results of the hypotheses tested are summarized in Tables 4, 5 and 6. Findings showed that US employees (*M* = 3.08, *SD* = 1.02) who were sexually harassed had greater job satisfaction than their Pakistani counterparts (*M* = 3.12, *SD* = .77) indicating that Pakistanis had higher job dissatisfaction, supporting H4.

**Table 4:** **Analysis of variance summary**

Study 1	U.S./Pakistan		
Sexual harassment			
Work outcomes	***F***	***Eta*** ^***2***^	***p***
Turnover intentions	1.69	.02	.17
Absenteeism	5.45	.03	.02
Job satisfaction	6.24	.03	.01
*Study 2*			
Turnover intentions	1.69	.21	.0001
Absenteeism	4.03	.25	.0001
Job satisfaction	2.23	.16	.01

**Table 5:** **Summary of regression analyses for variables predicting job satisfaction (H1), turnover intentions (H2), absenteeism (H3), and indirect responses (H7) in order of hypotheses (N = 324)**

	Study 1			Study 2		
Variable	***B***	***SE B***	***β***	***B***	***SE B***	***β***
H1 Sexual harassment	−0.18	0.12	−0.15	−0.18	0.11	−0.14
H1 Education	0.11	0.07	0.17	−0.24	0.36	−0.05
H2 Sexual harassment	0.20	1.00	0.14	6.57	0.03	0.20
H2 Education	0.25	0.09	0.19	3.65	0.09	0.03
H3 Sexual harassment	0.15	0.10	−0.03	−2.49	0.08	−0.03
H3 Education	0.42	0.10	0.06	0.19	0.25	0.06
H7 Country	0.24	0.11	0.24	−0.59	0.12	−0.38
H7 Education	0.02	0.07	0.03	−1.33	0.06	−0.02

**Table 6:** **Correlations**

		Country	Education	SH scale	Absenteeism	Turnover	Job sat
Country	Pearson correlation	1	.157	-.021	.536**	.358**	-.082
	Sig. (2-tailed)		.055	.799	.000	.000	.316
Education	Pearson correlation	.157	1	.026	.009	.022	-.085
	Sig. (2-tailed)	.055		.750	.910	.789	.300
SH Scale	Pearson correlation	-.021	.026	1	-.012*	.185**	-.131
	Sig. (2-tailed)	.799	.750		.888	.024	.111
Absenteeism	Pearson correlation	.536**	.009	-.012*	1	.606**	.047
	Sig. (2-tailed)	.000	.910	.888		.000	.565
Turnover	Pearson correlation	.358**	.022	.185**	.606**	1	-.135
	Sig. (2-tailed)	.000	.789	.024	.000		.098
Job sat	Pearson correlation	-.082	-.085	-.131	.047	-.135	1
	Sig. (2-tailed)	.316	.300	.111	.565	.098	

**Correlation is significant at the 0.01 level (2-tailed).

*Correlation is significant at the 0.05 level (2-tailed).

In study 2, findings showed that US employees (*M* = 3.28, *SD* = .85) who were sexually harassed had higher job satisfaction than their Pakistani counterparts (*M* = 3.33, *SD* = .70) indicating that Pakistanis had higher job dissatisfaction, also supporting H4.

### Sexual harassment, country, education, and turnover intentions

H5, that employees experiencing workplace sexual harassment will have higher turnover intentions in Pakistan (*M =* 3.15, *SD =* .75) than in the US (*M =* 2.58, *SD =* 1.37) was not significant on the univariate level (as detailed in Table 4) and, therefore, was not substantiated by Study 1. However, in Study 2, employees experiencing workplace sexual harassment significantly had higher turnover intentions in Pakistan (*M* = 3.15, *SD* = .75) than in the US (*M* = 2.41, *SD* = 1.53) on the univariate level showing support for H5 (see Table 4).

### Sexual harassment, country, education, and absenteeism

H6 posited that employees experiencing sexual harassment will have higher overall absenteeism in Pakistan (*M =* 3.16, *SD =* .76) than in the US (*M =* 1.97, *SD =* 1.36) was supported. There were similar significant results in Study 2. Overall absenteeism in Pakistan (*M* = 3.17, *SD* = .70) was higher than in the US (*M* = 1.88, *SD* = 1.17). Also, see Table 4.

### Sexual harassment and indirect strategies

Regression analysis results (*t*(211) = 6.59, *p* = .0001) showed that country significantly predicted that targets from Pakistan (*M* = 3.30; *SD* = .56) would be more likely to respond to sexual harassment indirectly than targets in the US (*M* = 2.94; *SD* = .82), supporting H7. Country also explained a significant proportion of variance in indirect scores (*R*^*2*^ = .06, *F*(2, 211) = 7.19, *p* = .001). The education control variable was not significant.

Study 2 regression results (*t*(162) = − 4.96, *p* = .0001) showed that country significantly predicted that targets from Pakistan (*M* = 3.30; *SD* = .64) would be more likely to respond to sexual harassment indirectly than targets in the US (*M* = 2.78; *SD* = .82), supporting H7. Country also explained a significant proportion of variance in indirect scores, *R*^*2*^ = .06, *F*(2, 162) = 12.70, *p* = .0001. The education control variable was also not significant in Study 2.

## Discussion

### Implications

This study examined whether the experience of sexual harassment impacts job satisfaction, turnover, and/or absenteeism in the US and Pakistan and distinguishes the different cultural approaches taken in response to being harassed. The results of this study show that the hurdle of workplace sexual harassment impacts on productive work environments in both Pakistan and the US even though the workplace environments are culturally distinct. In particular, the effects of sexual harassment in both samples increased the likelihood employees would have greater overall turnover intentions, overall absenteeism, and job dissatisfaction.

There are a number of differences between the climate in the US workplace and the climate in the Pakistani workplace. For example, because the relationship between cultural values and workplace outcomes is stronger in culturally tight societies such as Pakistan, and is weaker in such culturally loose societies as the US (Taras et al. 2011), it would take a much greater effort to affect a change in the Pakistani workplace with regard to sexual harassment than it would in the US which is a loose and more fluid culture.

Additionally, the high power distance present in Pakistan makes outside opposition to tacitly accepted sexual harassment practices by those in authority to be perceived as principally disrespectful. On the other hand, those in the low-power-distant US workplace are more likely to be able effect changes with regard to sexual harassment because the authority structure in the US is based to a larger extent on legitimate power (Raven and French 1958). Consequently, the law rules and those breaking the law are more likely to be formally prosecuted than in Pakistan where the laws are there but not enforced (Akhtar and Métraux 2013; Nosheen 2011; Qureshi 2013) because of paternalistic face considerations.

However, in most communities, women are considered an important factor in achieving rural development goals and are half of the manpower needed for rural development (Bozorgmanesh and Sadighi 2011). As a result, for economic development to thrive, women’s active participation in developing enterprises needs to be encouraged (Shaikh et al. 2011). Yet, women who are sexually harassed are less likely to participate in the workforce (Bernstein et al. 1992) and are more likely to leave their jobs (Sims et al. 2005). Therefore, the negative individual consequences of sexual harassment can have financial implications for the organizations who absorb the costs of productivity declines, absenteeism, impaired health, and turnover (Faley et al. 1999). Thus, organizations in all cultures need to be vigilant about sexual harassment (Cortina and Wasti 2005).

#### Sexual harassment and overall job satisfaction

Gruber (2003) suggests that sexual harassment’s effects are universal. Moreover, several researchers believe that the negative consequences of sexual harassment extend beyond individual nations to include multicultural organizations (DeSouza and Solberg 2003). Globalization has magnified this issue because of increasingly multicultural workplaces. Consequently, there is a need to understand differing belief systems guiding professional intercultural workplace conduct.

Overall, H1 was confirmed. Specifically, sexual harassment, regardless of the country, has a negative effect on employee job satisfaction. Findings also updated US findings in previous research indicating that sexual harassment leads to lower job satisfaction e.g., (Fitzgerald et al. 1999). Finally, low job satisfaction is important because it has been shown to act as an antecedent to turnover intentions (O’Connell and Korabik 2000) and to be inversely related to actual turnover (Carsten and Spector 1987).

#### Sexual harassment and turnover intentions

US studies suggest that sexual harassment is positively associated with turnover intentions (Shupe et al. 2002; Sims et al. 2005). In fact, meta-analyses by Steel and Ovalle (1984) show that turnover intentions are the strongest predictor of turnover. Moreover, turnover is one of the most indicative behavioral variables representing organizational decline because turnover is an indicator of dysfunctions in the overall organizational system (Knowles 1976). Likewise, in Pakistan a case study conducted with university teachers indicated that personal factors such as sexual harassment are the most significant issue in their turnover intentions (Ali Shah et al. 2010).

In turn, this study supported H2 that employees experiencing versus not experiencing sexual harassment have greater turnover intentions. While sexual harassment leads to turnover intentions, this study also indicated that whether people are educated also affects turnover intentions. Since educated workers tend to have more employment options, they are more likely to contemplate leaving their job when they are sexually harassed. This is a particular problem for businesses that would like to retain an educated workforce. It is, therefore, imperative for organizations to recognize that sexual harassment policies are necessary to retain employees.

#### Sexual harassment and overall absenteeism

While people may be unable to leave their job for financial reasons after experiencing sexual harassment, they may feel compelled to take off from work occasionally due to stress (Rospenda et al. 2005). Job withdrawal is a sign of a desire to leave one’s job and often precedes quitting or retirement (Hanisch et al. 1998). Experiencing sexual harassment in the US has been found to be significantly related to job withdrawal (Gruber 2003; Sims et al. 2005) and absenteeism (Baba et al. 1998). In Pakistan, Shahzad et al. (2011) found that job stress and job dissatisfaction predicts absenteeism.

Despite these findings, this study’s results only partially supported the notion in H3, that employees experiencing versus not experiencing sexual harassment will have greater overall absenteeism. Results were significant without education in the model. However, adding education caused sexual harassment to drop out as a predictor of absenteeism, leaving education as the sole predictor. Thus, overall, employees with higher education are more likely to respond to sexual harassment with absenteeism. As a result, employers should have sexual harassment policies in place, particularly with a more educated workforce for lower absenteeism.

#### Sexual harassment, job satisfaction, and absenteeism

H4 and H6, indicating that employees experiencing sexual harassment would have lower job satisfaction and higher absenteeism in Pakistan than in the US, was supported. While Pakistani employees may not be able to leave their jobs due to financial constraints, when sexually harassed, the stress may be just too great not to take off from work. The costs associated with sexual harassment are important for employers to take into account, particularly in developing Pakistan. These findings expand our understanding of global sexual behaviors at work by including the work experiences of Pakistanis. Understanding that the issue of sexual harassment is worse in Pakistan is critical in developing informed harassment policies and in guiding employer efforts to prevent harassment through training and awareness-raising campaigns. Moreover, given the prominence of Pakistan in world news and its strategic relationship with the US, both employers and workers can benefit from a more informed view of Pakistan and its civil society.

#### Sexual harassment and indirect strategies

This study showed that employees from Pakistan respond less directly to sexual harassment than employees from the US, supporting H7. In keeping with characteristics of collectivistic and high power-distant cultural attributes, Pakistani women tend to avoid bringing shame upon their family that could be caused by reporting sexual harassment (Savitha 2010). Current attitudes that sexual harassment is not a serious social issue in Pakistan (Morley et al. 2005) and associated collectivistic paternal values in Pakistan militate against targets being able to stand up and to demand human rights at work. Besides a lack of support mechanisms, Pakistani targets of sexual harassment are effectively ignored and kept silent (Morley et al. 2005).

On the other hand, Pakistanis tend to manage sexual harassment with subtle, indirect, and nonverbal forms of communication (Chan et al. 1999). This is because such tactics do not violate collectivistic, high power-distant, and paternalistic values which shun direct confrontations. Thus, it is important for policy makers to understand that Pakistanis prefer to address sexual harassment issues indirectly.

### Future research

This study reports on sexual harassment in the US and an understudied population, Pakistan. As a developing country, it is important that Pakistan maintains a viable workforce. However, the effects of sexual harassment result in negative work outcomes. Pakistan is similar to the US in that both populations experience lower job satisfaction, absenteeism, and turnover intentions in response to sexual harassment. However, in some cases in this study, the education covariate was significant. As a result, future research is needed to determine the impact education has on workplace outcomes independent of sexual harassment to a greater extent.

Finally, past research in the US takes a direct and legal approach to the problem of sexual harassment. However, in Pakistan, data indicates that a more indirect response to sexual harassment is preferred. Future research is needed to explore different indirect solutions to sexual harassment so that policies incorporating such strategies can be established and implemented.

Harassed targets, particularly from collectivistic cultures, tend to handle their circumstances using indirect strategies rather than making assertive public claims and objections. If organizations wish to employ a global workforce they will need to develop managerial strategies appropriate to the cultural values of their host country employees. Future research is needed to test out possible managerial strategies to determine which are most successful.

Given the increasing internationalization of business, it is vital to be aware of cultural differences in the interactions between men and women in organizations. It is also possible that commonplace employee overseas assignments in multinational corporations will cause employees to have to contend with cultural values and sensitivities much different from the ones in the host country. With a better grasp of this process, organizations may be in better positions to intervene.

### Limitations and conclusion

Given limitations in available participants for this study, convenience samples were used. Limitations are also tied in with our small samples, self-report data, and cross-sectional analysis. In addition to alternative designs addressing these limitations, future research should look toward longitudinal analyses of corporate effects due to organizational cultures that tolerate sexual harassment. This study adds to the sexual harassment literature by presenting a cross-cultural variability analysis including a previously unexamined culture and sexual harassment effects on work outcomes including job satisfaction, absenteeism, and turnover.

Understanding how the work outcomes of sexually harassed employees are impacted by cultural factors is important in a world of interconnected economies for at least two reasons. First, enlightening multinational businesses on the potential complications of interactions between employees from different countries could be helpful in strategic planning efforts. Moreover, this knowledge could be used in cross-cultural training programs tailored to Pakistani employees. Appropriate and targeted sexual harassment training based on cultural factors and the particular needs and sensitivities of employees would be more effective than training based on the flawed assumption that employees have similar backgrounds and values.

Second, the increased awareness from intercultural sexual harassment research could help guide and encourage multinational corporations to apply sexual harassment policies appropriately across different cultures. Identifying countries and cultures where there is a higher likelihood for employees to be sexually harassed and where there is a greater tolerance of sexual harassment incidences is critical because in certain countries it may be necessary to apply more vigorous and frequent communication of sexual harassment policies to employees.
